# Age-related neuroimmune signatures in dorsal root ganglia of a Fabry disease mouse model

**DOI:** 10.1186/s12979-023-00346-8

**Published:** 2023-05-12

**Authors:** Jeiny Luna Choconta, Verena Labi, Cristiana Dumbraveanu, Theodora Kalpachidou, Kai K. Kummer, Michaela Kress

**Affiliations:** 1 Institute of Physiology, Medical University of Innsbruck, Innsbruck, Austria; 2 Institute of Developmental Immunology, Medical University of Innsbruck, Innsbruck, Austria

**Keywords:** Lysosomal storage disorder, DRG, Sensory neurons, Macrophages, Inflammation, Phagocytosis, Immune cells

## Abstract

Pain in Fabry disease (FD) is generally accepted to result from neuronal damage in the peripheral nervous system as a consequence of excess lipid storage caused by alpha-galactosidase A (α-Gal A) deficiency. Signatures of pain arising from nerve injuries are generally associated with changes of number, location and phenotypes of immune cells within dorsal root ganglia (DRG). However, the neuroimmune processes in the DRG linked to accumulating glycosphingolipids in Fabry disease are insufficiently understood.

Therefore, using indirect immune fluorescence microscopy, transmigration assays and FACS together with transcriptomic signatures associated with immune processes, we assessed age-dependent neuroimmune alterations in DRG obtained from mice with a global depletion of α-Gal A as a valid mouse model for FD. Macrophage numbers in the DRG of FD mice were unaltered, and BV-2 cells as a model for monocytic cells did not show augmented migratory reactions to glycosphingolipids exposure suggesting that these do not act as chemoattractants in FD. However, we found pronounced alterations of lysosomal signatures in sensory neurons and of macrophage morphology and phenotypes in FD DRG. Macrophages exhibited reduced morphological complexity indicated by a smaller number of ramifications and more rounded shape, which were age dependent and indicative of premature monocytic aging together with upregulated expression of markers CD68 and CD163.

In our FD mouse model, the observed phenotypic changes in myeloid cell populations of the DRG suggest enhanced phagocytic and unaltered proliferative capacity of macrophages as compared to wildtype control mice. We suggest that macrophages may participate in FD pathogenesis and targeting macrophages at an early stage of FD may offer new treatment options other than enzyme replacement therapy.

**Supplementary Information:**

The online version contains supplementary material available at 10.1186/s12979-023-00346-8.

## Introduction

Lysosomal storage disorders (LSD) comprise a group of approximately 70 rare, inheritable monogenetic diseases related to lipid metabolism [1]. Fabry disease (FD) is considered the most common LSD, with a prevalence of 1:3′100 - 1:1′400 births [2–4]. FD is caused by one or more of at least 941 known mutations of the X-chromosome linked lysosomal hydrolase alpha-galactosidase A (α-Gal A) [5], which cleaves terminal alpha-galactosyl residues from glycosphingolipids and glycopeptides to degrade these macromolecules [6]. Dysfunctional α-Gal A causes the accumulation of glycolipids, such as globotriaosylceramide (Gb3), lyso-globotriaosylceramide (lyso-Gb3), B glycolipid antigen, P1 antigen, and digalactosylceramide, which cannot be degraded by other mechanisms [7]. As a consequence, glycolipids aggregate within the lysosomes, thereby forming lamellar-shaped inclusions [8], that mainly affect the cardiac system and the vasculature [9–11], the kidneys [12, 13], as well as the peripheral and central nervous system. Gb3 accumulates in the peripheral and central nervous system, specifically in neurons of sensory ganglia, Schwann cells, endothelial cells, and pericytes of endoneurial capillaries [14, 15]. Small fiber neuropathies and pain are common features of FD already in early life, which progress and diversify over time with increasing age of the patients [16–26]. While the pain in FD is generally accepted to arise from neuronal damage in the peripheral nervous system with small-fiber neuropathy, loss of neuronal terminals [27] and a dysregulation of sodium channels [28], injection of Gb3 induces local pain and enhances voltage-gated Ca^2+^ currents that lead to increased intracellular Ca^2+^, causing neurotoxic effects [29, 30]. Thus, extracellular glycosphingolipids, such as Gb3, might directly affect nociceptive primary afferents and contribute to FD-associated neuropathic pain [29].

Several transgenic mouse models have been developed to study FD pathophysiology [31–35]. All models display abnormalities resembling human FD, including Gb3 accumulation in the peripheral nervous system, alterations in sensorimotor function, hyposensitivity to painful stimuli, as well as upregulation of the transient receptor potential cation channel subfamily V member 1 (TRPV1) and voltage-gated sodium (Nav1.8) channels [15, 25, 27, 36, 37]. FD with a global depletion of α-Gal A on a C57BL/6 (B6) genetic background [35, 36, 38, 39] in contrast to FD mice on B6/129 background exhibit less prominent cardiac and renal symptoms as well as less profound alterations in thermal sensation [40, 41].

Signatures of neuropathic pain are generally associated with neuroimmune alterations and changes of the number and phenotypes of immune cells within the dorsal root ganglia (DRG) [42]. For example, immune cells invade the space between the neuronal cell soma and the covering satellite cell layer after nerve injury, and this is generally accepted to be of relevance for neuropathic pain pathogenesis [43, 44]. However, the link between accumulating glycosphingolipids and specific immune related pathologies has not been sufficiently addressed so far.

In order to obtain novel insight into FD-related deficits and a possible involvement of neuroimmune dysfunction in the peripheral nervous system, we explored lysosomal trafficking as well as macrophage morphology and phenotypes within the DRG. We discovered signatures of pathologically increased phagocytic activity that may be causally related to FD small fiber neuropathies. Alternatively, macrophage involvement may be indicative of compensatory resolving measures to minimize the neuronal damage that is caused by the aberrant accumulation of toxic lipid species.

## Material and methods

### Mouse model

Male α-galactosidase A^-/0^ (GlaKO^-/0^), female heterozygous GlaKO^+/−^, (kindly provided by Dr. A. Kulkarni, National Institute of Health, NIDCR, Bethesda, USA [35]) and male or female wildtype (wt; i.e., Gla^+/0^ or Gla^+/+^, respectively) littermates were used in this study. Animals were housed under specific pathogen-free (SPF) conditions at constant room temperature of 24 °C on a 12 h light/dark cycle with lights on from 07:00 to 19:00 and ad libitum access to autoclaved pelleted food and water. All experiments were performed in accordance with the Ethics Guidelines of Animal Care (Medical University of Innsbruck), as well as the European Communities Council Directive of 22 September 2010 on the protection of animals used for scientific purposes (2010/63/EU), and approved by the Austrian Bundesministerium für Bildung, Wissenschaft und Forschung (permit number BMWF-66.011/0148-V/3b/2019).

### Indirect immune fluorescence microscopy

Male and female animals were deeply anesthetized using a mix of xylazine 10 mg/kg (Xylasol 8-00178, LIVISTO) and ketamine 100 mg/kg (Ketasol 8-00173, LIVISTO) and transcardially perfused with room temperature Dulbecco’s Phosphate Buffered Saline (PBS, 14190-144, Gibco), followed by ice-cold 4% paraformaldehyde in PBS (PFA; J19943, Thermo Scientific). DRG were postfixed overnight (4% PFA) and subsequently embedded in 4% agarose. Sections of 50 µm were cut on a Leica S1000 vibratome and stored in PBS with 0.05% sodium azide until further processing.

Sections were washed three times for 5 min each with PBS and PBS containing 0.25% Triton X-100 (PBST) and blocked with 4% normal serum from donkey or goat in PBST for 90 min. After incubation with primary antibodies in blocking solution overnight at 4 °C, sections were washed three times with PBST and incubated with secondary antibodies in blocking solution for 1 h. Sections were washed with PBST and PBS three times for five minutes, incubated with DAPI (1:10′000) for 10 min, mounted on object slides and embedded in Mowiol 4–88 (Roth). For IBA1, CD68, and CD206 detection, heat-induced antigen retrieval (Sodium citrate buffer at 80 °C, 450 rpm for 30 min) was performed prior to immunostaining [45].

DRG cell preparations (see below) were fixed in 4% PFA for 30 min at room temperature. Fixed cells were washed three times for 5 min each with PBS containing 0.2% Bovine serum albumin and 0.2% Triton X-100 (PBS/BSA/Triton). Cells were incubated in 5% normal goat serum in PBS/BSA/Triton for 30 min. Primary antibodies were diluted in PBS/BSA/Triton and incubated overnight at 4 °C. Following three washes for 5 min each with PBS/BSA/Triton, cells were incubated with secondary antibodies for 1 h at room temperature. The final wash step was performed with PBS/BSA/Triton and PBS (three times for 5 min each). Cells were stained with DAPI (1:10′000) for 10 min, mounted on object slides and embedded in Mowiol 4–88 (Roth).

The following antibodies and concentrations were used: anti-CD77 (rat monoclonal, Beckman Coulter IM0175, 1:100), anti-IBA1 (goat polyclonal, Novus Biologicals NB100-1028, 1:100), anti-CD68 (rat monoclonal, Abcam ab53444, 1:1000), anti-CD206 (goat polyclonal, R&D systems AF2535, 1:100), anti-Tuj1 (rabbit polyclonal, Abcam ab18207, 1:1000), anti-LAMP1 (rabbit monoclonal, Cell Signalling 9091, 1:100), anti-Rat IgG (goat polyclonal, Thermo Fisher, Alexa Fluor™ 594 A11007, 1:500), anti-goat IgG (donkey polyclonal, Thermo Fisher, Alexa Fluor™ 594 A11058, 1:500), anti-rabbit IgG (goat polyclonal, Thermo Fisher, Alexa Fluor™ 594 A11012, 1:1000). In order to evaluate the specificity of the immunostaining, DRG sections were incubated with the same solutions as described above but without the primary antibodies (negative control). Negative controls did not show unspecific staining.

### Cell culture and transwell chemotaxis assay

Murine BV-2 cells were grown in RPMI-1640 medium (21875034, Gibco) supplemented with 10% fetal bovine serum (FBS, 10106169, Gibco) and 1% Penicillin/Streptomycin (PenStrep 15140122, Gibco). Cells were passaged every 3–4 days for a maximum of 30 passages. 24 h before the experiment, 4 × 10^5^ cells/mL were cultured in RPMI-1640 medium supplemented with 2 mM L-Glutamine (25030149, Gibco) and 1% Penicillin/Streptomycin. Lyso-Gb3 (Avanti Polar lipids, INC) and Gb3 (02446–91, Nacalai Tesque INC) was prepared as 1 mM stock solution in DMSO (Dimethyl sulfoxide, D4540, Sigma-Aldrich). For cell migration experiments, the stock solution was dissolved in FBS free media at 0.1 and 1 μM and added to the lower chamber of the transwell permeable supports (3464, Costar). BV-2 cells (5 × 10^4^ in 100 μl) resuspended in FBS free culture media were added to the upper chamber and allowed to migrate through the polycarbonate membrane of the transwell system (6.5 mm in diameter with 8 μm pore size) for 24 h at 37° C (humidified atmosphere of 5% CO_2_). Cells that did not migrate but stayed on the upper surface of the filter were scraped off, whereas cells that had migrated to the lower surface were fixed in 4% PFA for 30 min at room temperature and stained with DAPI (1:10′000), imaged and counted using Imaris 9.7.0 software (Bitplane, UK).

### Preparation of single DRG cell suspension

Male and female mice (20 - 26 weeks old) were deeply anesthetized using a mix of xylazine (10 mg/kg) and ketamine (100 mg/kg), and transcardially perfused with ice-cold PBS followed by tissue collection. DRG were harvested and transferred into Dounce buffer (15 Mm HEPES, 0.6% (w/v) Glucose in Hank’s Balance Salt Solution, H8264, Sigma). Homogenization of the DRG tissue was performed with a loose pestle in the Dounce homogenizer 15–20 times (Tenbroeck homogenisator, 10198611, DWK Life Science). The homogenate was filtered using a sterile 70 μm nylon cell strainer into a sterile 50 mL conical tube. The resulting single-cell suspension was centrifuged at 800 × g for 10 min at 4 °C and the pellet resuspended in FACS buffer (PBS with 2% (v/v) fetal bovine serum).

### Flow cytometry

DRG single-cell suspensions were blocked for non-specific antibody-binding with anti-CD16/32 Fc-Block (BioLegend 101320, 1:100) and incubated in an antibody cocktail consisting of anti-CD163-PE (BioLegend 156703, 1:200), anti-TLR4-PE/Cy7 (BioLegend 117609, 1:200), anti-CD206-PerCP/Cy5.5 (BioLegend 141715, 1:200), anti-CD11b-APCCy7 (BioLegend 101225, 1:200), anti-CD68-Alexa700 (BioLegend 137025, 1:200), anti-CD80-Bv421 (BioLegend 104725, 1:200), anti-CD86-Bv510 (BioLegend 105039, 1:200), anti-Gr1-Bv605 (BioLegend 10844, 1:200), anti-F4/80-Bv785 (BioLegend 123141, 1:200), and anti-CX3CR1-FITC (BioLegend 149019, 1:200) for 20 min at 4 °C. Exclusion of dead cells was achieved by resuspending cells in FACS buffer containing 10 nM TO-PRO-3 (Invitrogen, Carlsbad, CA, USA, T3605). Flow cytometry from the samples was performed using a BD LSRFortessa device and BD FACSDiva Software (BD Biosciences). Raw data were analyzed using the *CytoExplorer* R package version 1.1.0 [46].

### Protein–protein interaction analysis

A previously published microarray mRNA expression profiling dataset for DRG from male GlaKO vs wt mice (20–24 weeks) was used for analyses [38]. Significantly regulated immune-related mRNAs were extracted and analyzed using STRING Database v. 11.5 and clustered using an interaction score of 0.4 and MCL clustering algorithm (inflation parameter: 3).

### Phagocytic activity

To determine phagocytic activity, 50′000 BV-2 cells/well were seeded (100 µL per well) in a 96 well plate (Nunclon Micro well 96-well, 167008, Thermo Scientific) and incubated in 5% FBS medium (RPMI-1640, 21875–034, Gibco) with 0.1 µM, 1 µM or 10 µM Lyso-Gb3 or Gb3 and/or 10 µM Cytochalasin D (PHZ1063, Invitrogen) over night at 37 °C (humidified atmosphere with 95% air and 5% CO_2_). pHrodo BioParticles (100 µL of 0.5 mg/mL, A10010, Invitrogen) were added to each well and incubated for 2 h at 37 °C. pHrodo uptake was measured using an Infinite M200 Pro microplate reader (TECAN) using 560 nm and 585 nm as excitation and emission wavelengths, respectively. Multiple reads per well were acquired in bottom mode, 16 flashes and 150 gain. Normalized relative fluorescence units (RFU) were obtained by subtracting the average fluorescence intensity of the no-cell negative control and normalizing against the added-Cytochalasin D control per condition.

### Viability assay

The viability test was performed as described previously [47]. Briefly, 2 × 10^5^ BV-2 cells/mL were seeded per well in a 24 well plate for 24 h in 10% FBS medium. Medium was changed to the treatment conditions (0.01, 0.1, 1, 10 µM Lyso-Gb3 or Gb3, 1% DMSO as vehicle) and incubated for 24 h in 5% FBS medium. Dilution 1:1 of trypan blue (0.4% Trypan Blue Solution, 93,595, Sigma-Aldrich) and cell suspension was prepared to count the viable (unstained) and nonviable (stained) cells. Triplicates were counted per condition.

### Preparation of short-term neuron cultures and lysosome tracking

Lumbar DRG from 40 weeks old male mice were harvested, cleaned and placed in DMEM medium (21885025, Gibco) with 0.05 mg/mL gentamicin (G1397, Sigma). DRGs were incubated with 0.09 mg/mL Liberase (05466202001, Roche) for 1 h, washed with PBS, and incubated with Trypsin–EDTA (1X, 25300054, Gibco) for 15 min. Cells were dissociated with a fire-polished Pasteur pipette in TNB-100® Medium (F8823, Biochrom) supplemented with 2.5% Protein-Lipid-Complex (F8820, Biochrom), 2 mM L-Glutamine and 1% Penicillin/Streptomycin, and centrifuged in a BSA density gradient at 500 rpm for 10 min (3.5% BSA in TNB-100® Medium with 2.5% Protein-Lipid-Complex, 2 mM L-Glutamine, 1% Penicillin/Streptomycin). The resulting pellet was resuspended in medium (TNB-100® supplemented with protein-lipid complex, L-glutamine and 1% Penicillin/Streptomycin) and further centrifuged at 760 rpm for 5 min. The pellet was resuspended in modified TNB-100 medium containing 2.5% Protein-Lipid-Complex, 2 mM L-Glutamine, 1% Penicillin/Streptomycin and 100 ng/mL 2.5S mNGF (N-100, Alomone Labs). Cells were seeded on glass cover slips coated with a mixture of 1% v/v Poly-L-Lysine (P4707, Sigma) and 10% v/v Laminin (L-2020, Sigma) and cultivated at 37 °C in 5% CO_2_ for 24 h. For lysotracker experiments, cells were incubated with 100 nM LysoTracker (Green L-7526, Invitrogen) at 37 °C in 5% CO_2_ for 45 min. Time lapse images were acquired every 2 s for 10 min on an Olympus IX71 inverted fluorescence microscope equipped with an Orca Flash 4.0 (C13440 Hamamatsu) with 60 × objective using excitation 504 nm and emission 511 nm (LedHUB, Omicron-Laserage Laserprodukte). Lysosomal trafficking analysis was performed using the “tracking spots” tool in Imaris 9.7.0 software (Bitplane, UK).

### Image analysis and quantification

Z stack images of tissue sections were acquired using a Leica TCS SP8 multiphoton (MP) microscope and processed by the Imaris 9.7.0 software (Bitplane, UK). Microscope settings were consistent between experiments and conditions (MP wavelength 795 nm, intensity 2%, gain 100%, offset 50%). Gb3 accumulation was calculated based on the total volume of anti-CD77 immune fluorescence and normalized against the volume of the image. Quantification of DRG macrophages was based on anti-IBA1 immunopositive cells. Filament and surface 3D reconstruction was used to analyse the morphology of DRG macrophages. Images of DAPI positive nuclei of migrated BV-2 cells were acquired from centric 9 optic fields using a Zeiss Axiovert 200 M microscope with a 20 × 0.3 Ph1 objective (1006–591, Zeiss). DRG neuron culture images were acquired using a Zeiss Axio Imager Z1 microscope with 100 × 1.46 oil immersion objective (420,792–9800, Zeiss).

### Statistical analysis

All statistical analyses were performed using RStudio v1.1.463 (R v3.6.0). Data were tested for normal distribution using Shapiro-Wilk normality test. For normally distributed data, Student’s t-test or two-way ANOVA followed by Bonferroni post-hoc test was performed. For data with skewed distribution, Mann–Whitney U-test or Kruskal–Wallis test was applied, followed by pairwise comparisons using Wilcoxon rank sum exact test and Bonferroni correction. Statistical tests used in the study are indicated in the figure legends. Values are given as mean ± standard error of the mean (SEM). Significance level was set to *p* < 0.05.

## Results

### Age-dependent Gb3 accumulation, soma enlargement and lysosomal dysfunction of GlaKO sensory neurons

**Fig. 1:**
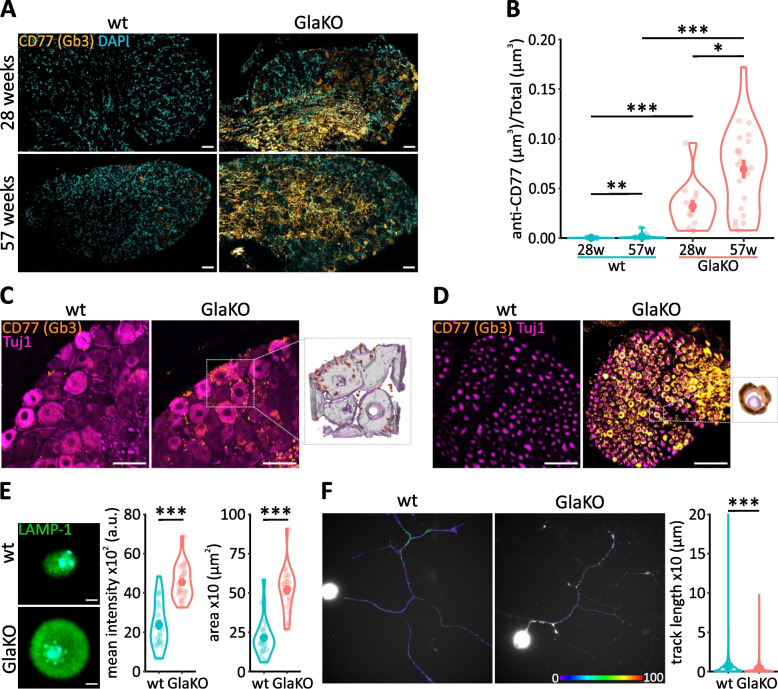
Age-depended glycosphingolipid accumulation and lysosomal dysfunctions in male GlaKO mouse DRG. **A** Gb3 was identified using indirect immune fluorescence microscopy using anti-Gb3 (CD77) in DRG from adult (28 weeks) and old (57 weeks) mice. Scale bar 50 µm. **B** Quantification of Gb3 accumulation. 28 weeks wt mice *n* = 6, GlaKO mice *n* = 7, 57 weeks wt mice *n* = 6, GlaKO mice *n* = 8. Statistical significance was assessed using Kruskal–Wallis test followed by pairwise comparisons using Wilcoxon rank sum exact test and Bonferroni correction. **C** 3D reconstruction of MP detail images from neuronal cell bodies and (**D**) axon bundles, showing in detail the localization of Gb3 depositions. Scale bar 10 µm. **E** Representative images of LAMP1 staining and quantification of increased lysosomal distribution and cell body area of isolated DRG neurons. Scale bar 5 µm. *n* = 22 cells per group. **F** Time-lapse analysis of lysosome trafficking in DRG neurons and track length measurements. *n* = 20 cells per group. Mann–Whitney U test. Values are given as mean ± SEM.* *p* < 0.05, ** *p* < 0.01, *** *p* < 0.001, if not otherwise stated

Like FD patients, GlaKO mice develop Gb3 accumulation in the peripheral nervous system accompanied by sensory deficits [15, 40, 48–52]. In line with these reports, we detected strikingly more Gb3 deposits in the DRG (Fig. 1A) of male 28 weeks old GlaKO as compared to wt mice (Fig. 1B; wt: 0.00022 ± 0.000075 a.u., vs. GlaKO: 0.032 ± 0.0061 a.u., *p* < 0.001). In addition, Gb3 accumulation was significantly enhanced in DRG old male GlaKO mice (Fig. 1B; 57 weeks, wt: 0.0011 ± 0.00048 a.u. vs. GlaKO: 0.069 ± 0.0093 a.u., *p* < 0.001) and to a lesser degree in female heterozygous Gla^/^^-^ and homozygous Gla^-/-^ mice (Additional file 1: Fig. S1A, B). Quantification of Gb3 indicated age-related progressive accumulation also in wt male mice (Fig. 1B; 28 weeks: 0.00022 ± 0.000075 a.u., vs. 57 weeks: 0.0011 ± 0.00048 a.u. *p* < 0.01), suggesting that Gb3 deposition is positively correlated with aging, and this was severely aggravated in FD indicative of premature aging. Gb3 deposition was mainly detectable in neuronal cell bodies and axon bundles of male GlaKO mice (Fig. 1C, D). Based on the mainly intracellular location of Gb3 in neurons together with the known enlargement of neuronal cell bodies in mouse and human FD DRG sections. [7, 53, 54], we queried further whether the enlargement of DRG neuron somata was maintained in vitro where extracellular Gb3 deposits were largely absent due to the culturing conditions. Size, morphology (Fig. 1E; wt: 215 ± 25.6 µm^2^ vs. GlaKO: 519 ± 30.1 µm^2^, *p* < 0.001) and in particular lysosomal structures were severely altered in FD neurons in vitro, as indicated by altered immunoreactivity for the lysosomal protein LAMP1 (Fig. 1E; wt: 2373 ± 246 a.u. vs. GlaKO: 4545 ± 188 a.u., *p* < 0.001). Since this could be related to alterations in the mobility of lysosomes, we loaded cultured neurons from male GlaKO mice and control littermates with lysotracker dye to quantify endo/lysosome movement, in vitro. Lysosomal displacement was significantly decreased in sensory neurons obtained from GlaKO compared to control mice (Fig. 1F; wt: 6.7 ± 0.077 µm vs. GlaKO: 3.7 ± 0.05 µm, *p* < 0.001), indicating severely hampered endo/lysosomal trafficking that may contribute to the sensory deficits associated with the disease.

### Immune biological processes in DRG

**Fig. 2:**
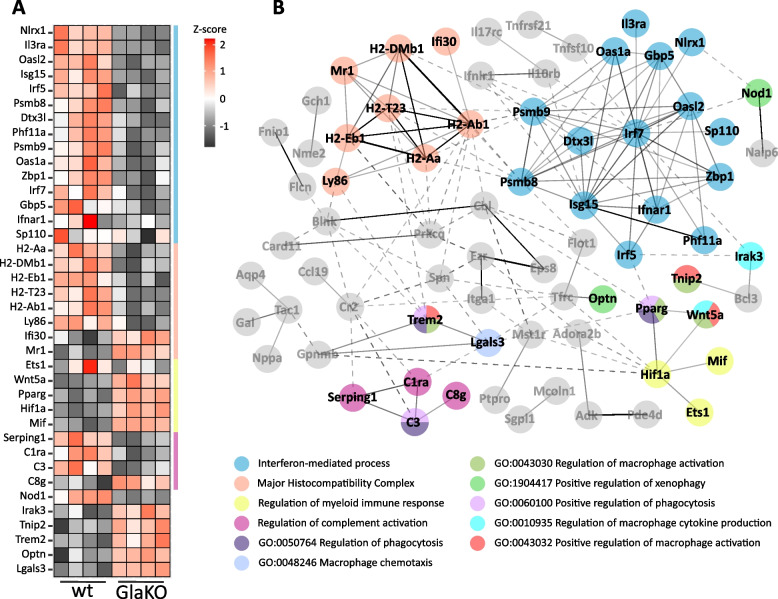
Immune interaction network of significantly regulated immune-related mRNAs obtained from a published microarray dataset [38] of male GlaKO DRG. **A** Heatmap depicting significant differentially expressed immune-related genes between control and GlaKO DRG of 20–24 weeks old male mice: interferon-mediated process, major histocompatibility complex, regulation of myeloid immune response, regulation of complement activation and genes related to macrophage activation and phagocytosis. **B** Protein–protein interaction network using STRING Database v. 11.5 and MCL clustering algorithm (inflation parameter: 3). Solid lines represent associations with high edge confidence and dashed lines represent lower confidence interactions

In addition to direct Gb3 induced neuronal deficits, immune cells resident in the DRG or invading from the vascular system can contribute to overall neuropathic changes associated with FD, and numerous deregulated functional clusters have emerged from our transcriptomic analysis of male mouse DRG [38]. Re-analysis of our data set with a particular focus on neuroimmune processes revealed that 95 out of the 812 differentially expressed genes were associated with immune processes (Fig. 2A, B); protein–protein interaction (PPI) and enrichment analyses revealed protein clusters associated with interferon processes, major histocompatibility complex, regulation of myeloid immune response and complement activation. In addition, genes related to macrophage activation and phagocytosis were overall upregulated in male GlaKO mice, such as interleukin 1 receptor associated kinase 3 (Irak3), TNFAIP3 interacting protein 2 (Tnip2), triggering receptor expressed on myeloid cells 2 (Trem2), optineurin (Optn) and galectin 3 (Lgals3). These findings support a disease-related deregulation of immune cell function that so far has not been addressed in detail.

### Morphology and phenotype of myeloid cells was altered in DRG of GlaKO mice

**Fig. 3:**
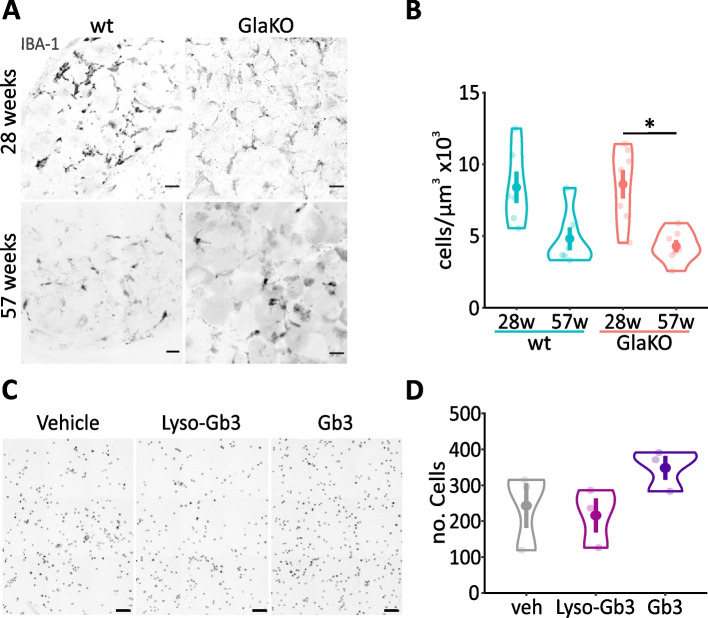
Unaltered number of macrophages in male GlaKO DRG supported by the no chemoattractant effect of Gb3 or Lyso-Gb3. **A** Representative images of DRG stained for anti-IBA1. Scale bar 50 µm. **B** Quantification of DRG macrophages. 28 weeks wt mice *n* = 6, GlaKO mice *n* = 7, 57 weeks wt mice (*n* = 6), GlaKO mice (*n* = 8). Statistical significance was assessed using Kruskal–Wallis test followed by pairwise comparisons using Wilcoxon rank sum exact test and Bonferroni correction. *p < 0.05. **C** Representative images of migrated BV-2 cells using the transwell assay, in vitro. Scale bar 100 µm. **D** In line with the in vivo data, 1 µM Lyso-Gb3 or 1 µM Gb3 did not significantly increase cell migration of BV-2 cells (0.1% DMSO as vehicle). Wells per condition *n* = 3. Statistical significance was assessed using Kruskal–Wallis test

In order to explore whether increased numbers of immune cells such as macrophages invading into the DRG could contribute to the increased DRG volume and the transcriptomic immune-related dysregulations in GlaKO DRG, we performed indirect immune fluorescence imaging using IBA-1 as a monocytic marker to quantify macrophages, in DRG sections. An age-dependent decline of macrophage numbers was evident in particular in male GlaKO (Fig. 3A; 28w: 8611 ± 988 cells/µm^3^ vs. 57w: 4289 ± 414 cells/µm^3^, *p* < 0.05) but also in wt mice. However, total numbers of macrophages were comparable in both genotypes indicating that Gb3 did not act as a chemoattractant for macrophages (Fig. 3A). This finding was reflected by results from a trans-well migration assay to assess the chemoattractant properties of Lyso-Gb3 and Gb3 using the monocyte cell line BV-2, in vitro. After a long-term exposure (24 h), a significant chemoattractant effect of Lyso-Gb3 or Gb3 was not evident (Fig. 3B). Of note, Lyso-Gb3 or Gb3 did not affect the viability of BV-2 cells (Additional file 1: Fig. S1C).

**Fig. 4:**
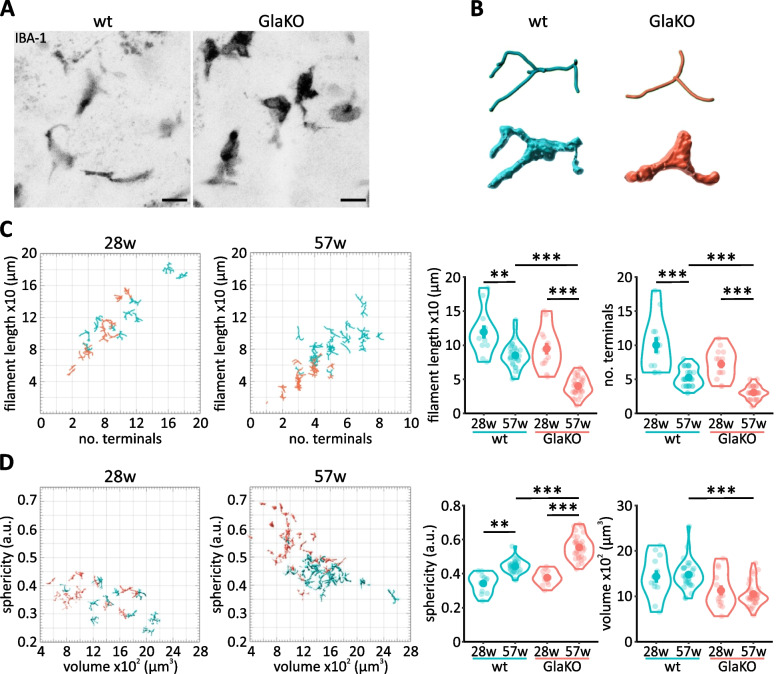
Age-depended morphological alteration of male GlaKO DRG macrophages. **A** Representative images of macrophage morphology based on IBA-1 staining. Scale bar 10 µm. **B** Representative 3D reconstruction of GlaKO DRG macrophages compared to control. **C** Filament tracing and **D** surface reconstruction of macrophages from 28 and 57w old mice. Between 12 and 30 cells per condition were analyzed using Imaris 9.7 with Filaments and Surfaces tools. Statistical significance was assessed using Kruskal–Wallis test followed by pairwise comparisons using Wilcoxon rank sum exact test and Bonferroni correction. Values are given as mean ± SEM. * *p* < 0.05, ** *p* < 0.01, *** *p* < 0.001, if not otherwise stated

More remarkably, the morphology of DRG macrophages was strikingly altered in GlaKO mice compared to wt littermates (Fig. 4A, B). 3D reconstruction of filament conformation and surface occupation showed that old GlaKO DRG macrophages were significantly less ramified (Fig. 4C; wt: 5.26 ± 0.245 terminals vs. GlaKO: 3.06 ± 0.162 terminals, *p* < 0.001) with a more rounded shape (Fig. 4D; wt: 0.44 ± 0.007 a.u. vs. GlaKO: 0.55 ± 0.012 a.u., *p* < 0.001), indicative of a potentially hyperactive functional state.

**Fig. 5:**
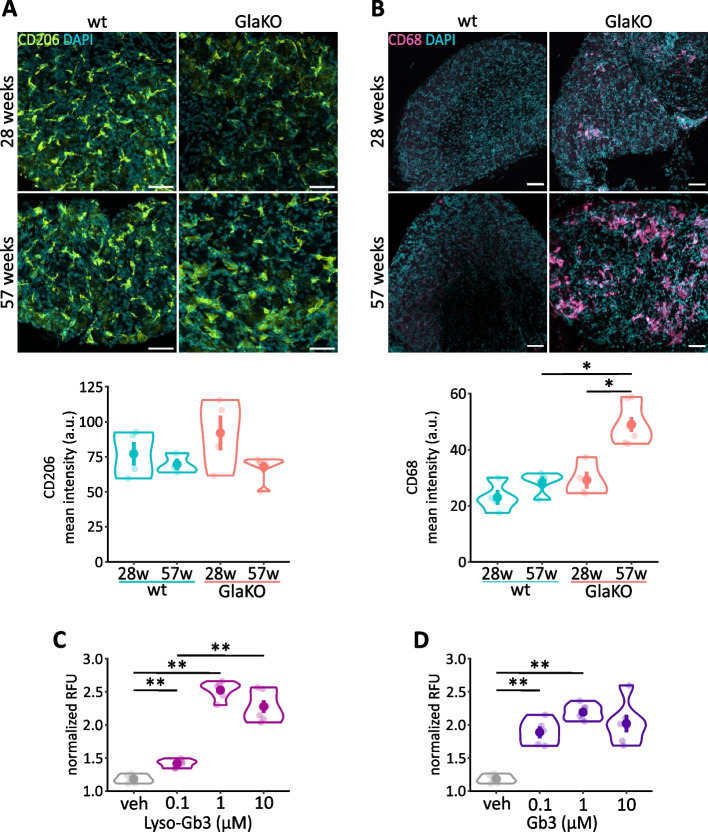
Increased macrophage CD68 expression indicative of enhanced phagocytic activity, in vitro. **A** MP images representing unaltered CD206 expression, M2 macrophages, in 28w and 57w GlaKO mice. Scale bar 50 µm. Male: 28 weeks wt mice *n* = 4, GlaKO mice *n* = 4, 57 weeks wt mice *n* = 4, GlaKO mice *n* = 6. **B** Aged GlaKO mice showed a significant upregulation of the endo/lysosomal marker CD68 in DRG. Scale bar 50 µm. Male: 28 weeks wt mice *n* = 4, GlaKO mice *n* = 4, 57 weeks wt mice *n* = 4, GlaKO mice *n* = 7. **C** Lyso-Gb3 or (**D**) Gb3 activated phagocytosis of BV-2 (1% DMSO was used as vehicle). Wells per condition *n* = 6. Statistical significance was assessed using Kruskal–Wallis test followed by pairwise comparisons using Wilcoxon rank sum exact test and Bonferroni correction. Values are given as mean ± SEM. * *p* < 0.05, ** *p* < 0.01, if not otherwise stated

To further investigate FD related alterations of macrophages, we analyzed the expression of specific markers to identify different macrophage phenotypes. CD206, mannose receptor C type 1 (MRC1) is expressed by M2 macrophages [55] and the number of CD206^+^ macrophages are decreased in GlaKO mice [50]. However, the numbers of monocytic cells showing CD206 labelling in the DRG were similar in male GlaKO and wt littermates (Fig. 5A). Furthermore, the endo/lysosomal marker CD68 enriched in macrophages belongs to the lysosomal-associated membrane protein (LAMP) family of glycoproteins and rapidly shuttles between the plasma membrane and endosome [56–58]. CD68 is expressed in cells of the mononuclear phagocyte lineage including macrophages, microglia, osteoclasts, and myeloid dendritic cells (DCs) and is upregulated in inflammation [58, 59]. Interestingly, significantly increased age-dependent expression of CD68 was evident in DRG from male GlaKO mice (Fig. 5B; wt: 28.3 ± 2.09 a.u. vs. GlaKO: 49 ± 2.63 a.u., *p* < 0.01) as well as female heterozygous Gla^+/-^ and homozygous Gla^-/-^ mice (Additional file 1: Fig. S1A). This finding suggested enhanced lysosomal activity as a consequence of increased phagocytosis in FD DRG macrophages.

Since phagocytosis may be a measure to remove excess amount of lipids such as Gb3, we evaluated phagocytic activity in the BV-2 monocytic cell line in vitro with pH-sensitive pHrodo BioParticles. BV-2 cells developed significantly increased phagocytic activity when exposed to 1 μM Lyso-Gb3 (Fig. 5C; vehicle: 1.18 ± 0.025 normalized RFU vs. Lyso-Gb3: 2.52 ± 0.053 normalized RFU, *p* < 0.01) or 1 µM Gb3 (Fig. 5D; vehicle: 1.18 ± 0.025 normalized RFU vs. Gb3: 2.19 ± 0.049 normalized RFU, *p* < 0.01). However, evidence for intracellular Gb3 accumulation was not detected in these cells and this is in line with previously described locations of Gb3 deposits in DRG either within neuronal cell bodies or Schwann cells or in the extracellular space. This supports the idea that other lipid products or cellular debris may be taken up and removed by phagocytosing macrophages as reported for other LSDs [60]. As a consequence, FD macrophages may secrete toxic materials via exosomes that can damage neighbouring neurons and aggravate neuropathic alterations resulting from FD [61].

### Specific myeloid cell populations are deregulated in male GlaKO mice

**Fig. 6:**
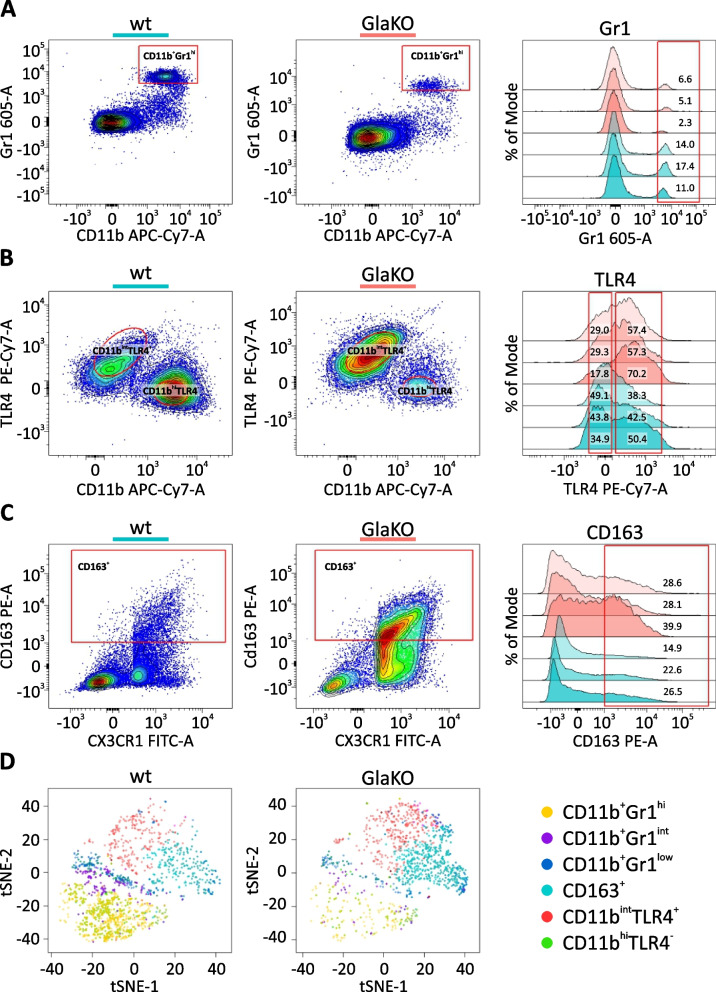
Reduced CD11b^+^Gr1^high^ and increased CD163^+^ CX3CR1^+^ and/or CD11b^+^ cell populations in male GlaKO DRG. **A** Flow cytometry analysis of myeloid cells in GlaKO and control DRG cell suspensions from 20 - 24 weeks old male mice. Representative scatter plots and histograms of CD11b and Gr1 evidencing a reduced fraction of CD11b^+^Gr1^high^ cells in GlaKO DRG. **B** Higher TLR4 expression and (**C**) enriched CD163^+^ CX3CR1^+^ and/or CD11b^+^ cells in GlaKO as compared to control DRG. **D** Dimensional reduction using tSNE and clustering analysis from each myeloid cell population identified in (**A**), (**B**) and (**C**). Pooled samples from 3 mice, wt male samples *n* = 3, GlaKO male samples *n* = 3

Further activation markers in the myeloid cell population in DRG were explored in single cell suspensions from male and female GlaKO and wt DRG prepared by physical homogenization for flow cytometry analysis (Fig. 6 and Additional file 1: Fig. S3). A gating strategy was established based on single-antibody control. Following doublets and dead cell exclusion (Additional file 1: Fig. S2), we assessed the leukocyte surface antigens CD11b and Gr1 and identified three subpopulations of monocytes/macrophages (CD11b^+^Gr1^−^, CD11b^+^Gr1^int^ and CD11b^+^Gr1^high^). GlaKO DRG showed a significant reduction of the CD11b^+^Gr1^high^ population as compared to wt control DRG (Fig. 6A). This cell population acts as an important suppressor of e.g. obesity-associated inflammation [62], suggesting that the depletion of CD11b^+^Gr1^high^ monocytes could possibly promote inflammatory processes in FD DRG. Systemic inflammation is initialized by increased toll like receptor 4 (TLR4) expression [63, 64], and we found TLR4 upregulated in fractalkine receptor CX3CR1^+^ and/or CD11b^+^ cells in GlaKO mice (Fig. 6B). Furthermore, the scavenger receptor cysteine-rich CD163 was enriched in both male GlaKO and female Gla^+/-±^ DRG (Fig. 6C and Additional file 1: Fig. S3C). In the spinal cord, CD163 is induced by electrical activity of C-nociceptors indicating microglia activation, and may have protective effects rather than promote inflammation [65]. We therefore assessed the myeloid cell populations from wt and GlaKO mice by tSNE dimensionality reduction [66]. tSNE plot confirmed expression enrichment of CD163^+^ (Fig. 6D, cyan cluster) and reduced CD11b^+^Gr1^high^ cell population in GlaKO DRG (Fig. 6D, yellow population). Collectively, these data revealed complex pro-inflammatory functional states of monocytes/macrophages in GlaKO DRG which may develop as a consequence of potential excitotoxic neuronal damage by Gb3 [29].

## Discussion

In line with previous reports of Gb3 deposited in neurons, skin and liver tissue in FD mice [67, 68], we found extracellular Gb3 accumulation accompanied by significant morphological alterations and an increased phagocytic phenotype in macrophages in the DRG of FD mice.

FD pathogenesis involves major biological processes including a chronic inflammatory response, extracellular matrix remodelling, peptidase activity, cellular response to reactive oxygen species, and dysregulation of metabolic processes. This suggests that the deposition of Gb3 and deficits of lysosomal storage give rise to complex homeostatic alterations in various tissues, including the peripheral and central nervous system [69]. Lysosomes are considered the relevant site of excess lipid deposits in LSD [70]. LAMP proteins are predominantly located in the membrane of lysosomes, and critically contribute to phagocytosis, autophagy, and lipid transport [71]. LAMP1 is distributed among autophagic organelles, such as lysosomes, which serve as degradation compartments that support neuronal survival and function [70]. Although LAMP1 is routinely used as a lysosome marker, and LAMP1-positive organelles are often referred to as lysosomal compartments, LAMP1 localization may not be restricted to lysosomes, rather indicating areas of lysosomal interactions with other cellular organelles, such as autolysosomes, endosomes, multivesicular bodies, and multilamellar bodies [72]. Phagocytic cells from FD patients express higher levels of LAMP1 [73], FD axon tracts accumulate LAMP1-positive lysosomes and non-surprisingly, lysosomal trafficking is severely affected in FD [74–76].

An increased Gb3 load in peripheral neurons is associated with more intense FD pain and hypersensitivity of nociceptive neurons [50, 74]. Topical application of Lyso-Gb3 or Gb3 causes mechanical hypersensitivity in mice, and this is associated with complex changes of transduction and action potential generation resulting in excitation and sensitization of nociceptive primary afferents [29, 54]. With increasing age, the hypersensitivity reverses into hyposensitivity in FD patients [24, 77] and GlaKO mice [25, 36]. This may occur as a consequence of excitotoxic Gb3 actions or defective lysosomal trafficking in relevant neuronal tissue as observed in the current study. In line with these reports, we found significant FD related morphological changes in cultured FD sensory neurons and structural redistribution of LAMP1-positive regions within the cell soma, indicative of lysosomal trafficking deficits and possibly neuronal damage associated with FD.

The consequences of nerve damage in FD are complex, but usually involve signatures of sterile inflammation which is particularly important for the resolution of nerve damage and for regenerative processes [78]. Of note, LAMP1 expression is not only aberrantly increased in neurons but also in phagocytic cells from FD patients. This phenotype can be improved via intravenous administration of enzyme replacement therapy which is the most common treatment for FD [73]. Increased expression of LAMP1 contributes to the neuroinflammatory response in LSD progression [79, 80], pointing towards immune cells as emerging FD modifying contributors [81, 82]. In models of neuropathic pain, a high density of immune cells is observed as a result of early proliferation of ED1^+^ macrophages phagocytosing myelin followed by de novo invasion of macrophages [44]. In contrast, macrophage numbers per µm^3^ were unaltered in GlaKO DRG. However, we found strikingly altered macrophage morphology suggesting FD-specific immune responses distinct from the response to traumatic nerve injury. Upregulation of CD68 in macrophages, indicative of increased phagocytotic activity, raise the possibility that macrophages are involved in the removal of pathological lipids such as Gb3. However, Gb3 accumulation was exclusively evident in the extracellular space but not inside IBA-1-positive cells, suggesting that other substrates such as neuronal debris or myelin resulting from neurotoxic cell damage rather than Gb3 itself may be phagocytosed by macrophages [83, 84]. In GlaKO male and Gla^+/-^ female mice, CX3CR1^+^ and/or CD11b^+^ myeloid cells showed enriched expression of the anti-inflammatory marker CD163 in DRG. Macrophages expressing CD163 are usually involved in the resolution of inflammation by activating anti-inflammatory pathways [85, 86] or through transferring mitochondria to DRG neurons [87]. Since elevated levels of CD163 are not only obtained from plasma samples of FD patient but are also reported in Gaucher disease or endomyocardial biopsies from patients with myocardial diseases [88, 89] the higher expression of CD163 on CX3CR1^+^ and/or CD11b^+^cells may resemble a compensatory reaction to resolve accumulating lipids and cell debris common to LSDs.

## Conclusions

Together, our data not only confirm pathological accumulation of lipid species in FD sensory neurons but rather suggests that Gb3 may affect morphology, phagocytic function and phenotypes of the myeloid cell lineage in an age-dependent manner. These alterations may be causally involved in disease pathogenesis, and FD-related changes of monocytes may not only be relevant for cardiac and renal FD pathologies [9–12] but also for disease related deficits in pain processing. Targeting macrophages could therefore offer novel perspectives for FD treatments in addition to enzyme replacement therapy.

## Supplementary Information

**Additional file 1: Figure S1.** Indirect immune fluorescence microscopy in murine heterozygous Gla^+/-^ and homozygous Gla^-/-^ female DRG, and viability assay in BV-2 cells. (A) Representative MP images of female DRG using anti-Gb3 (CD77), anti-CD68 and anti-CD206. Scale bar 50 µm. (B) Quantification of stained DRG sections, 42 weeks Gla^+/-^ *n*=3 and Gla^-/-^ *n*=3 mice. Statistical significance was assessed using Mann–Whitney U test. (C) Viability test of BV-2 cells in Lyso-Gb3 and Gb3 (1% DMSO was used as vehicle). Values are given as mean ± SEM. **Figure S2.** Gating strategy. Following doublet and dead cell exclusion using TO-PRO3, we explored Gr1^+^ vs. CD11b^+^ cells populations (CD11b^+^Gr1^low^, CD11b^+^Gr1^int^, CD11b^+^Gr1^high^). Downstream analysis was performed based on CX3CR1^+^ and/or CD11b^+^ populations, which were gated from the “Live cells” gate. **Figure S3.** Flow cytometry analysis of myeloid cells in Gla^+/-^ and control DRG cell suspensions from 20 - 24 weeks old female mice. (A) Representative scatter plots and histograms of CD11b and Gr1. (B) TLR4 expression and (C) higher CD163 expression in CX3CR1^+^ and/or Cd11b^+^ in GlaKO. (D) Dimensional reduction using tSNE and clustering analysis from each identified cell population in (A), (B) and (C). Pooled samples from 3 mice. 20 - 26 weeks wt female samples *n*=3, Gla^+/-^ female samples *n*=4.

## Data Availability

The datasets used and/or analyzed during the current study are available from the corresponding author on request.

## Abbreviations

α-Gal A: Alpha-galactosidase A
CD11b: Integrin subunit alpha m
CD68: Macrosialin
CD163: Scavenger receptor cystein-rich
CD206: Mannose receptor C type 1
CX3CR1: CX3C motif chemokine receptor 1
DMSO: Dimethyl sulfoxide
DRG: Dorsal root ganglia
FBS: Fetal bovine serum
FD: Fabry disease
Gb3: Globotriaosylceramide
GlaKO: α-Gal knock-out mouse model
IBA1: Ionized calcium-binding adapter molecule 1
LAMP: Lysosomal-associated membrane protein
LSD: Lysosomal storage disorders
Lyso-Gb3: Lyso-globotriaosylceramide
MP: Multiphoton
PBS: Phosphate Buffered Saline
PenStrep: Penicillin/Streptomycin
PFA: Paraformaldehyde
TLR4: Toll like receptor 4
TRPV1: Transient receptor potential cation channel subfamily V member 1
wt: Wildtype
