# Supplementary figures and images for: Age-related neuroimmune signatures in dorsal root ganglia of a Fabry disease mouse model
Source: Immun Ageing. 2023 May 12;20:22. doi: 10.1186/s12979-023-00346-8 (PMC10176851; doi:10.1186/s12979-023-00346-8)

Figure S1

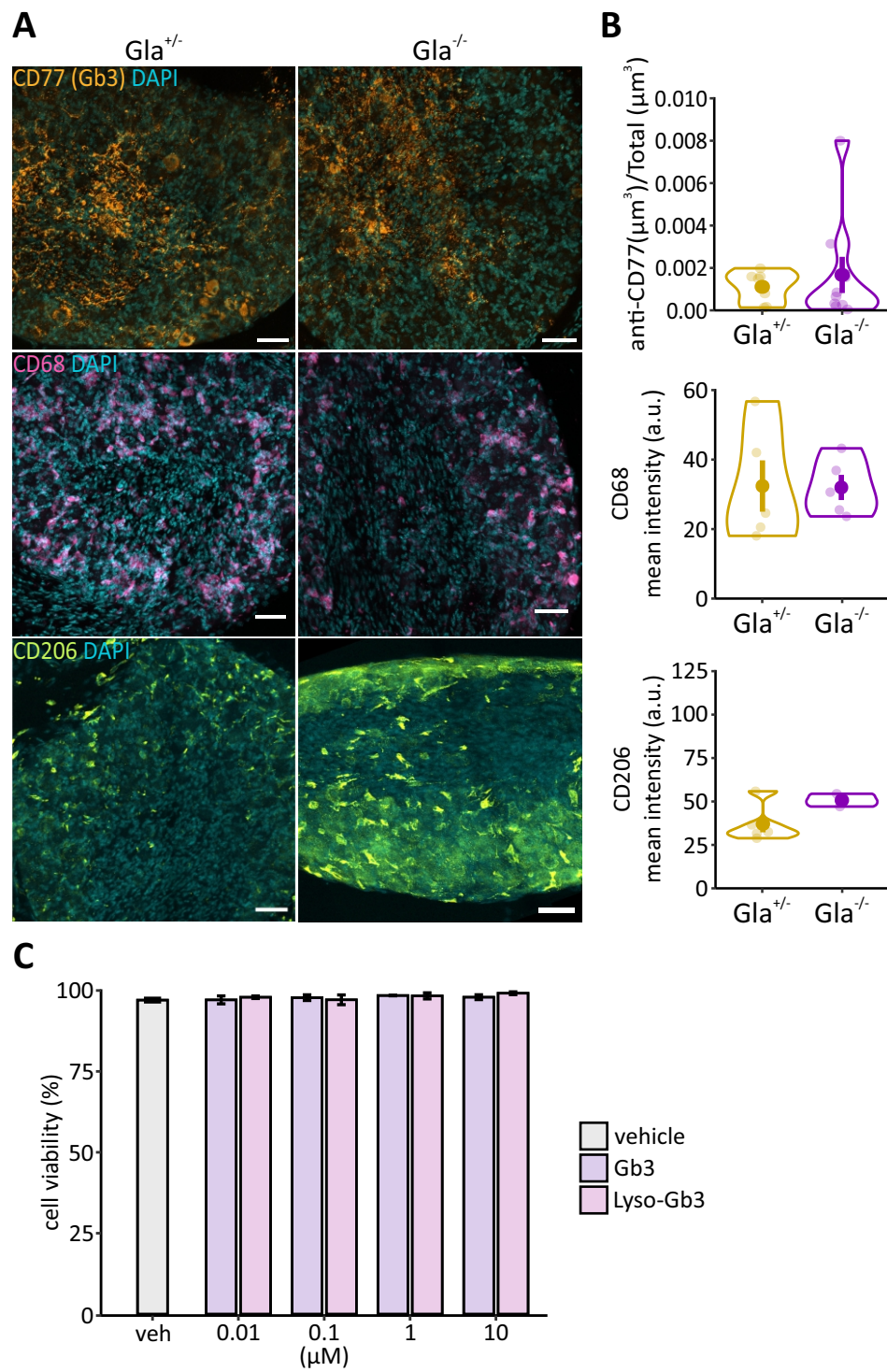

Figure S2

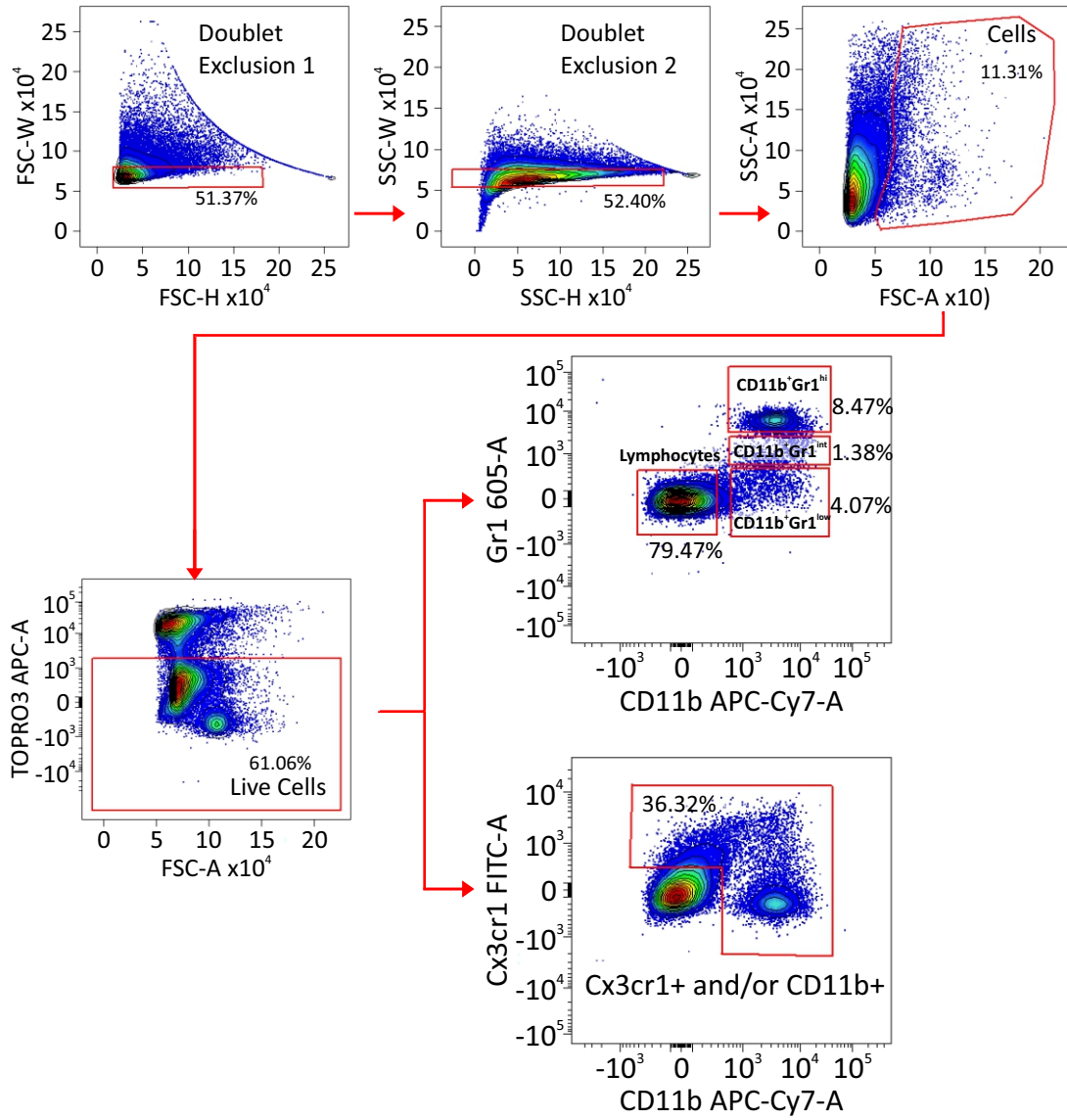

Figure S3

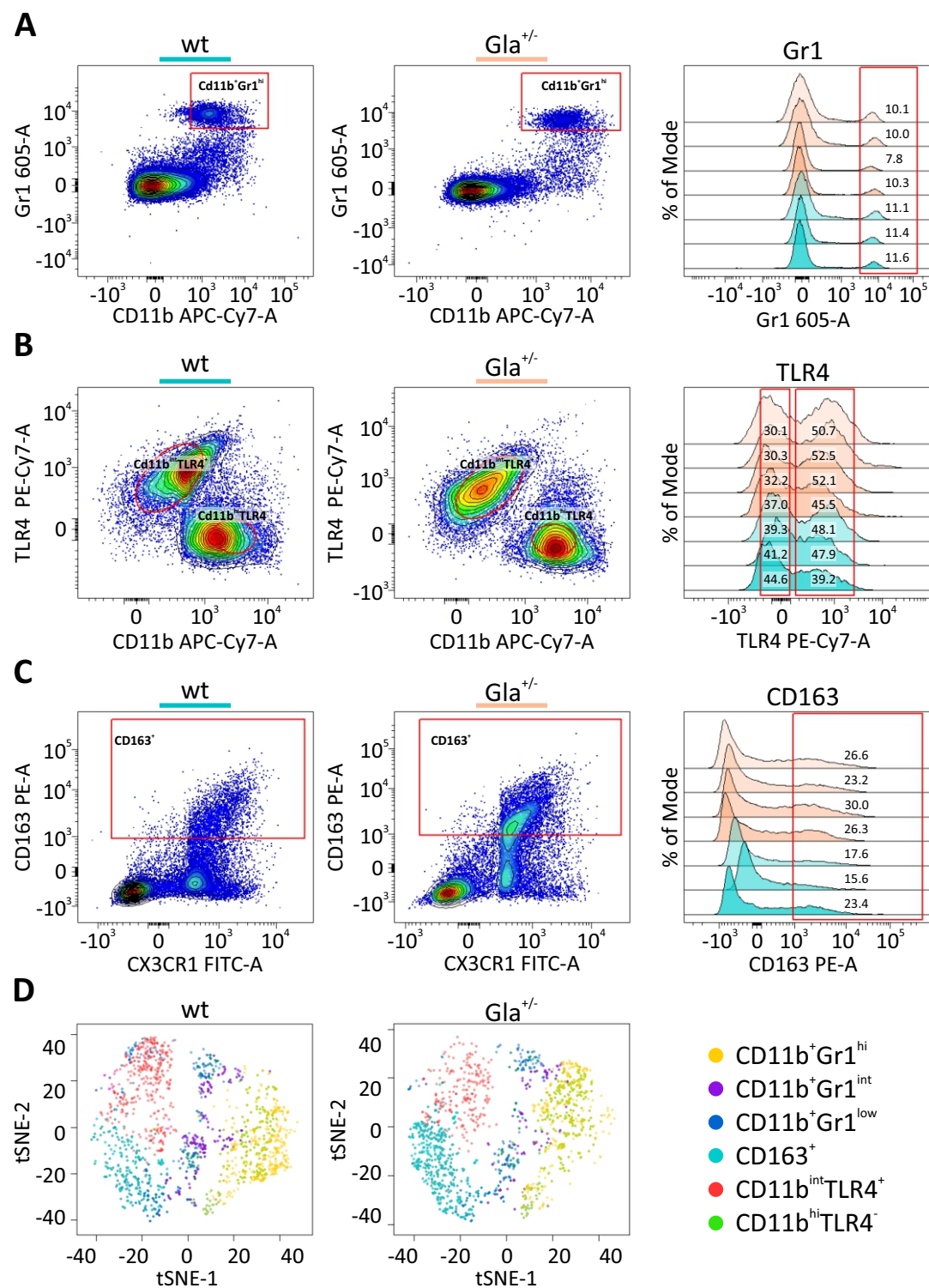

Supplement: Supplementary file 1 — Additional file 1: Figure S1. Indirect immune fluorescence microscopy in murine heterozygous Gla+/- and homozygous Gla-/- female DRG, and viability assay in BV-2 cells. (A) Representative MP images of female DRG using anti-Gb3 (CD77), anti-CD68 and anti-CD206. Scale bar 50 µm. (B) Quantification of stained DRG sections, 42 weeks Gla+/- n=3 and Gla-/- n=3 mice. Statistical significance was assessed using Mann–Whitney U test. (C) Viability test of BV-2 cells in Lyso-Gb3 and Gb3 (1% DMSO was used as vehicle). Values are given as mean ± SEM. Figure S2. Gating strategy. Following doublet and dead cell exclusion using TO-PRO3, we explored Gr1+ vs. CD11b+ cells populations (CD11b+Gr1low, CD11b+Gr1int, CD11b+Gr1high). Downstream analysis was performed based on CX3CR1+ and/or CD11b+ populations, which were gated from the “Live cells” gate. Figure S3. Flow cytometry analysis of myeloid cells in Gla+/- and control DRG cell suspensions from 20 - 24 weeks old female mice. (A) Representative scatter plots and histograms of CD11b and Gr1. (B) TLR4 expression and (C) higher CD163 expression in CX3CR1+ and/or Cd11b+ in GlaKO. (D) Dimensional reduction using tSNE and clustering analysis from each identified cell population in (A), (B) and (C). Pooled samples from 3 mice. 20 - 26 weeks wt female samples n=3, Gla+/- female samples n=4. [file 12979_2023_346_MOESM1_ESM.pdf]
